# Genomic diversity is similar between Atlantic Forest restorations and natural remnants for the native tree *Casearia sylvestris* Sw.

**DOI:** 10.1371/journal.pone.0192165

**Published:** 2018-03-07

**Authors:** João Paulo Gomes Viana, Marcos Vinícius Bohrer Monteiro Siqueira, Fabiano Lucas Araujo, Carolina Grando, Patricia Sanae Sujii, Ellida de Aguiar Silvestre, Mariana Novello, José Baldin Pinheiro, Marcelo Mattos Cavallari, Pedro H. S. Brancalion, Ricardo Ribeiro Rodrigues, Anete Pereira de Souza, Julian Catchen, Maria I. Zucchi

**Affiliations:** 1 Graduate Program in Genetics and Molecular Biology, University of Campinas, Campinas–SP, Brazil; 2 Universidade do Sagrado Coração, Bauru–SP, Brazil; 3 Graduate Program in Tropical and Subtropical Agriculture, Agronomic Institute of Campinas, Campinas–SP, Brazil; 4 Department of Genetics, “Luiz de Queiroz” College of Agriculture, University of São Paulo, Piracicaba-SP, Brazil; 5 Embrapa Pecuária Sudeste, Embrapa, São Carlos–SP, Brazil; 6 Department of Forest Sciences, “Luiz de Queiroz” College of Agriculture, University of São Paulo, Piracicaba-SP, Brazil; 7 Department of Biology, “Luiz de Queiroz” College of Agriculture, University of São Paulo, Piracicaba-SP, Brazil; 8 Department of Plant Biology, University of Campinas, Campinas–SP, Brazil; 9 Department of Animal Biology, University of Illinois at Urbana—Champaign, Champaign–IL, United States of America; 10 Agência Paulista de Tecnologia dos Agronegócios, Piracicaba–SP, Brazil; National Cheng Kung University, TAIWAN

## Abstract

The primary focus of tropical forest restoration has been the recovery of forest structure and tree taxonomic diversity, with limited attention given to genetic conservation. Populations reintroduced through restoration plantings may have low genetic diversity and be genetically structured due to founder effects and genetic drift, which limit the potential of restoration to recover ecologically resilient plant communities. Here, we studied the genetic diversity, genetic structure and differentiation using single nucleotide polymorphisms (SNP) markers between restored and natural populations of the native tree *Casearia sylvestris* in the Atlantic Forest of Brazil. We sampled leaves from approximately 24 adult individuals in each of the study sites: two restoration plantations (27 and 62 years old) and two forest remnants. We prepared and sequenced a genotyping-by-sequencing library, SNP markers were identified *de novo* using Stacks pipeline, and genetic parameters and structure analyses were then estimated for populations. The sequencing step was successful for 80 sampled individuals. Neutral genetic diversity was similar among restored and natural populations (A_R_ = 1.72 ± 0.005; H_O_ = 0.135 ± 0.005; H_E_ = 0.167 ± 0.005; F_IS_ = 0.16 ± 0.022), which were not genetically structured by population subdivision. In spite of this absence of genetic structure by population we found genetic structure within populations but even so there is not spatial genetic structure in any population studied. Less than 1% of the neutral alleles were exclusive to a population. In general, contrary to our expectations, restoration plantations were then effective for conserving tree genetic diversity in human-modified tropical landscapes. Furthermore, we demonstrate that genotyping-by-sequencing can be a useful tool in restoration genetics.

## Introduction

Increasing environmental degradation has led to the promotion of ecological restoration worldwide, particularly for forest ecosystems [1–3]. Forest restoration methods are selected based upon the degree of ecosystem degradation [4,5], the availability of native forest fragments to act as seed sources in the landscape [6,7] the goals of restoration interventions [4], and funding constraints [8,9]. For all restoration approaches, forest succession must proceed in time, which is the primary ecological process controlling the recovery of forest ecosystems [10]. However, several factors drive tropical forest succession. Although some of these factors are unpredictable and difficult to manipulate at the site level, such as dispersal by fauna, others can be controlled at some level during restoration planning, implementation, or adaptive management; these are the factors that receive special attention from practitioners. Genetic diversity of reintroduced populations is a key factor for the long-term persistence of native species in forest restoration sites that can be manipulated by practitioners, based on the selection of natural populations and seed-trees for producing planting stocks [9,11,12].

Genetic diversity is defined as the variety of alleles and genotypes in a population, which drives morphological, physiological and behavioral differences in individuals and populations [13]. It is important for the long-term viability of populations because of the influence on adaptive potential, inbreeding, and genetic drift [14–16]. Moreover, the risk of extinction of natural populations increases at reduced genetic diversity [16]. One of the causes of increased vulnerability is the limitation of evolutionary potential to respond to environmental changes [17], a critical issue in the Anthropocene [18]. Tree species, in particular, are highly vulnerable to reduced genetic diversity in founder populations, because of the lack of mobility and susceptibility to significant environmental changes within a lifetime [19]. In spite of the numerous conceptual arguments for considering genetic diversity in biodiversity conservation, a large gap occurs between theory and practice.

Forest restoration projects, for example, began long before the recognition of the importance of genetic diversity for their long-term sustainability [2]. The primary ecological goals have been the recovery of forest structure and woody species diversity, with less focus on genetic diversity. More recently, many studies have addressed the importance of genetic diversity for restoration success [11,12,20,21]. However, few studies have measured genetic diversity in restored areas, which limits our understanding of the genetics of restoration.

Forest restoration plantings are often implemented using seedlings from few mother trees, and the level of genetic diversity and kinship among planted seedlings has received little attention [22,23]. Populations reintroduced through restoration plantings may have low genetic diversity and be genetically structured due to founder effects and genetic drift, which limit the potential of restoration to recover ecologically resilient plant communities. These limitations are a concern because inbreeding can lead to reduced fertility and individual survival in some species. However, natural remnants located near forest restoration areas can conserve genetic diversity [24] and supply a diverse set of alleles for the population undergoing restoration, acting as stepping stones that promote genetic connectivity among forest fragments across human-modified landscapes [25,26]. Few studies have assessed genetic diversity following forest restoration interventions, particularly in older restoration projects, implemented before the recognition of the importance of genetic diversity for the long-term viability of plant populations. Consequently, the expectation is that populations established through restoration plantations will have low genetic diversity and be structured due to founder effects, as observed for regenerating palm populations in second-growth tropical forests [27].

The advent of short-read sequencing, which can assess large-scale sets of single nucleotide polymorphisms (SNPs), has eased the study of population genetics in a genomic scale using populations of non-model species [28], such as tropical trees. Additionally, SNPs offer the potential for large-scale scanning of the genome by providing a large number of markers that improve the accuracy of population genetic estimates [29]. Among the methodologies used for SNP discovery, Elshire et al. (2011) [30] proposed a method that uses restriction enzymes to reduce the complexity of the genome. This genotyping-by-sequencing (GBS) approach is a low cost, highly specific, and reproducible method to detect SNPs and can be applied in non-model species. Although each SNP represents a point measurement, we can combine these SNPs into a continuous distribution across the genome to conduct scans for genetic variation, which can be applied to native species [31], such as those used in forest restoration projects, and advance the knowledge on restoration genetics.

In this context, we assessed the genetic diversity and genetic structure of populations of the native tree *C*. *sylvestris* in restoration plantations and natural remnants of the Brazilian Atlantic Forest to verify if there are differences in genetic diversity level between these type of forest fragments.

## Materials and methods

### Study species

*Casearia sylvestris* Sw. (Saliaceae; *Salix* sp. and *Populus* sp. are tree species of this family widely used in population genetic studies in a genomic scale) is a pioneer tree species highly abundant in secondary forests [32–35], distributed from Mexico to Argentina [36]. The mating system has not been studied using molecular markers approach yet, but the flowers are hermaphroditic, and the species is self-compatible [37,38]. Similar to other species of this genus, *C*. *sylvestris* is usually pollinated by various species of flies, whereas seed dispersal occurs by small birds over short distances [39].

### Study areas and sampling

We performed the study in the Brazilian Atlantic Forest, a global hot spot for biodiversity conservation that is estimated to contain from 1% to 8% of the world's terrestrial species [40] and more than 8000 endemic species. However, less than 8% of the original area remains within continuous forests [41] and only 11–16% when secondary forests and small fragments (<100 ha) are considered [42]. We selected two natural remnants (Campinas and Tietê) and two forest restoration plantations (Cosmópolis and Iracemápolis) in São Paulo State, southeastern Brazil, for this study (S1 Appendix). The natural remnant in Campinas (Mata de Santa Genebra, hereafter SG, 22°49.36’ S, 47°6.60’ W) is 252-ha and is embedded within one of the largest urban centers in Brazil, in a landscape dominated by pastures, sugarcane plantations, and small-scale forestry. Tietê (hereafter TT, 23°0.11’ S, 47°43.66’ W) is a 70-ha forest fragment surrounded by extensive pastures, similar to most natural remnants of the Brazilian Atlantic Forest. Cosmópolis (hereafter CO, 22°40.30’ S, 47°12.25’ W) is a 25-ha forest restoration plantation implemented between 1955 and 1960 in the riparian buffer of the Jaguari River in a landscape matrix dominated by sugarcane plantations, with few natural fragments remaining. The Iracemápolis (hereafter IR, 22°34.60 S, 47°30.18’ W) study site is a 50-ha forest restoration plantation established between 1988 and 1990 at the border of a water reservoir that supplies drinking water to the city of Iracemápolis [9]. All four forests had a high abundance of *C*. *sylvestris* individuals, particularly near the borders and in the understory. The CO population was established with nursery-grown seedlings produced with seeds harvested in an old landscaping planting in the “Luiz de Queiroz” College of Agriculture of the University of São Paulo in Piracicaba–SP, whereas the IR population was established with nursery-grown seedlings bought from a forest nursery in Campinas–SP and another in São Paulo—SP. Leaves from approximately 24 adult individuals, separated by approximately 12 m, were collected from each of the four populations. Using GPS equipment, a minimum distance of 12 m was established between individuals, with a margin of error of a maximum of 4 m (S1 Dataset). In the forest restorations we tried to select individuals of the same generation, but as *C*. *sylvestris* is a rapid growing plant species, both planted (i.e., adult individuals growing in the original planting lines) and regenerating individuals (i.e., adult individuals growing in between the original planting lines) may have been sampled.

### SNP discovery and data analysis

#### Library preparation and sequencing

DNA was extracted from leaf tissue samples of individuals from each of the four populations following the method described by Doyle and Doyle (1987) [43]. To increase the chances of obtaining the same proportion of reads per individual, the samples with high quality DNA were diluted to the same concentration based on electrophoresis in agarose 1% gel. Preliminary tests with different restriction enzymes were performed. PstI digestion showed the highest concentration of fragments with the greatest distribution in size. The library was then prepared according to the method described by Elshire et al. (2011) [30], using barcodes with at least three nucleotides of difference to identify each individual. Finally, single-end sequencing of the 95 samples was performed in a single lane of an Illumina HiSeq 2000 (Illumina, San Diego, CA) producing sets of 100bp reads.

#### Data processing

Sequences of each sample were demultiplexed and cleaned using the *process_radtags* program from the *Stacks* package v. 1.44 [44]. A quality score filter (-q, *process_radtags*) was applied across each sample to retain only high-quality sequencing reads. Reads with any non-called base were discarded (-c, *process_radtags*). Barcodes were recovered when they had a maximum of one mismatch (-r, *process_radtags*). Because a reference genome is not available for *C*. *sylvestris*, the loci were built *de novo*. Sequences from each sample were aligned using a minimum depth of 3 and allowing up to 2 mismatches with the *ustacks* program (-m 3, -M 2, *ustacks*). Subsequently, a catalog of loci was built from subsets of samples, allowing 2 mismatches (-n 2, *cstacks*) between loci from distinct samples. All samples were matched against the catalog, using *sstacks*, to determine the set of loci in each. We then implemented a correction step with *rxstacks*, discarding loci when the mean log likelihood of the locus in the metapopulation was less than -10; confounded loci were also removed. Using the populations program, a filter was applied across loci and individuals to retain loci with a maximum of 20% missing data (S2 Dataset). To separate the outlier loci in the set of SNPs, we used an F_ST_-based approach to assess population pairwise comparisons using the infinite alleles model and a false discovery rate (FDR) of 0.05, as implemented in the *Lositan* software [45]. Subsequently, neutral candidate loci were separated from outlier loci using 99% confidence intervals boundaries. All below estimates were calculated using the set of neutral candidate loci (hereafter neutral loci).

#### Genetic diversity

To compare the genetic diversity in forest restoration populations and natural remnants, we used the following population parameters: number of different alleles, number of private alleles, observed heterozygosity, expected heterozygosity and the inbreeding coefficient (F_IS_). We tested for significant differences in heterozygosities and F_IS_ using confidence intervals calculated based on 1000 bootstrap resamples. Estimates of population genetic parameters were calculated using the *PopGenKit* [46] and *hierfstat* [47] packages. Venn diagrams were generated using the *VennDiagram* package [48]. All packages were developed for use in the *R* software [49].

#### Genetic differentiation and structure

To understand the partitioning of genetic diversity, we calculated overall and pairwise F-statistics using the *diveRsity* package [50]. The significance of these estimates was determined using 95% confidence intervals based on 1000 bootstrap resamples across individuals. A Discriminant Analysis of Principal Components (DAPC) was conducted to explore the genetic structure in these populations using *adegenet* [51,52].

#### Spatial genetic structure

The spatial genetic structure (SGS) analysis was performed based on the kinship coefficient proposed by Loiselle et al. (1995) [53]. This kinship coefficient is robust and unbiased by the frequency of rare alleles. The number and distance intervals of classes were defined to suit each population. To summarize the spatial genetic structure, we used the *Sp-statistic* proposed by Vekemans and Hardy (2004) [54], which is calculated as *S*_*p*_ *= - β*_*log*_ */ 1—F*_*1*_; where β_log_ is the regression slope of the kinship coefficient on the log of geographic distance, and *F*_*1*_ is the mean of the kinship coefficient between individuals estimated for the first distance class. For each distance class, the 95% confidence interval was obtained based on 1000 permutations. For all analyses of SGS within the populations, we used the *SPAGeDi* program developed by Hardy and Vekemans (2002) [55], whereas the spatial autocorrelation graphs were plotted with the *R* software [49].

## Results

### SNP discovery and data processing

Considering the high number of reads obtained (317,802,888 reads), the sequencing was successful. After cleaning, demultiplexing and filtering of missing data, 861 high-quality biallelic SNPs were identified, which were used to characterize the genetic diversity of *C*. *sylvestris* individuals distributed across the four populations. For these results, the mean sequencing depth per individual ranged from 1.25 to 43.40, whereas mean depth per locus ranged from 4.70 to 83.22 (SD = 8.58). After all filtering steps it was possible to retain 80 individuals for the subsequent analyses. Using the F_ST_ approach in pairwise population comparisons to detect outlier loci, we identified 662 neutral candidate loci and 199 outlier candidate loci.

### Genetic diversity

We found no significant differences in observed and expected heterozygosities and inbreeding coefficient between the natural remnants and forest restoration populations (Table 1).

**Table 1:** Genetic diversity parameters for *Casearia sylvestris* populations in natural remnants and restoration plantations in the Atlantic Forest of southeastern Brazil.

Pop.	N	A	P	H_O_	H_E_	F_IS_
CO	22	1217	2	0.13 (0.13–0.14)	0.17 (0.16–0.17)	0.19 (0.15–0.23)
IR	18	1213	1	0.14 (0.13–0.16)	0.17 (0.15–0.17)	0.15 (0.10–0.19)
SG	21	1223	4	0.14 (0.13–0.15)	0.17 (0.15–0.17)	0.14 (0.10–0.18)
TT	19	1216	2	0.13 (0.12–0.15)	0.16 (0.15–0.17)	0.17 (0.12–0.22)

CO–Forest restoration in Cosmópolis, SP; IR–Forest restoration in Iracemápolis, SP; SG–Natural remnant in Campinas, SP; TT—Natural remnant in Tietê, SP. n–Sample size; A–Number of different alleles; P–Number of private alleles; H_O_−Observed heterozygosity; H_E_−Expected heterozygosity; F_IS_−Within population fixation index.

**Fig 1:**
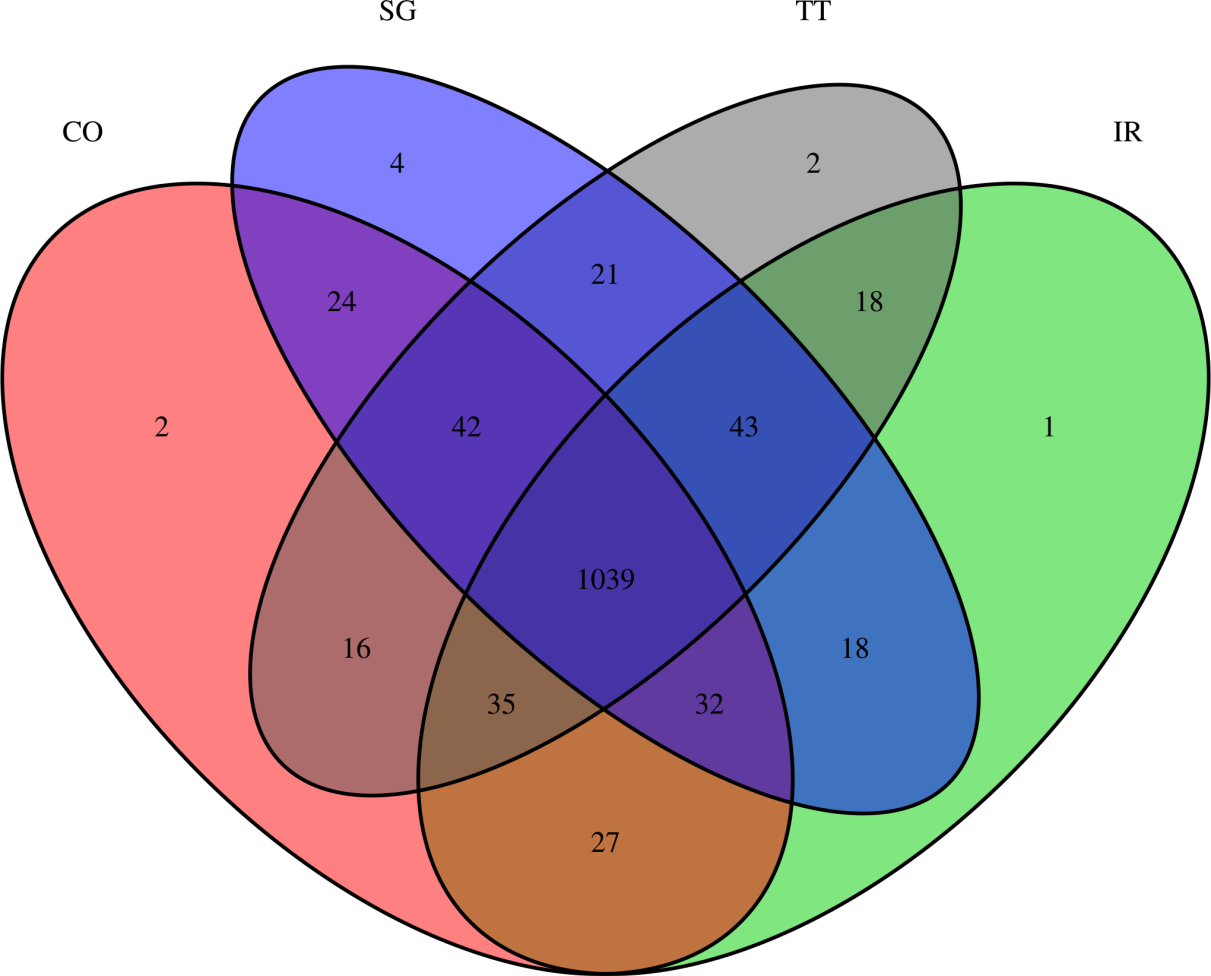
Venn diagram showing the number of shared alleles among groups of *Casearia sylvestis* populations from restoration plantations and natural remnants in the Atlantic Forest of southeastern Brazil. Seventy-nine percent of all alleles were found in all populations. CO–Samples from forest restoration in Cosmópolis, SP; IR–Samples from forest restoration in Iracemápolis, SP; SG–Samples from natural forest in Campinas, SP; TT–Samples from natural forest in Tietê, SP. Different colors represent each of the four studied populations.

Of the 1324 different alleles from neutral loci detected in the four populations, all populations shared approximately 79%, 11% corresponded to alleles shared by at least three populations, 9% were in at least two populations, and less than 1% were exclusive to a population (Fig 1). These results are consistent with those presented in Table 1, indicating no genetic differences among the forest restoration and natural remnant populations.

### Genetic differentiation and structure

**Fig 2:**
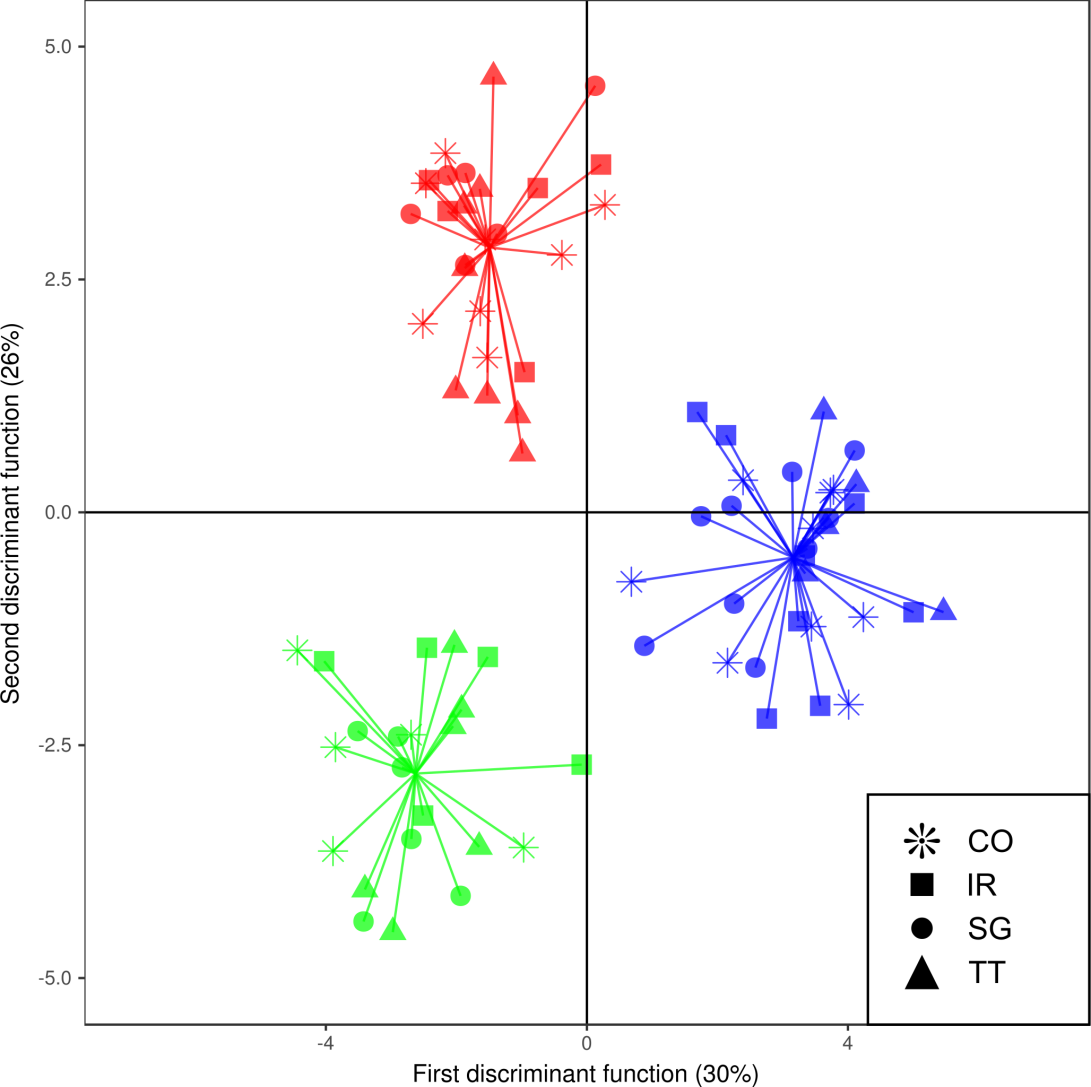
Scatterplot from DAPC showing the two discriminant functions for *Casearia sylvestis* populations from restoration plantations and natural remnants in the Atlantic Forest of southeastern Brazil. Different symbols represent populations from four studied forest fragments. Different colors represent tree different clusters found using BIC of DAPC. These clusters were built with samples from all pre-defined populations, indicating that genetic structure was not based on population subdivision. CO–Samples from forest restoration in Cosmópolis, SP; IR–Samples from forest restoration in Iracemápolis, SP; SG–Samples from natural forest in Campinas, SP; TT–Samples from natural forest in Tietê, SP.

The populations were not genetically differentiated. Moreover, all pairwise F_ST_ estimates (Table 2, upper triangular) were not significantly different from zero (P > 0.05). Based on these estimates, which consider the set of neutral loci, no population genetic structure was detected. Additionally, DAPC did not show any structure by pre-defined population subdivision (Fig 2).

**Table 2:** Pairwise Nei’s F_ST_ value (upper triangular. F_ST_ value were not significantly different from zero based on 95% confidence intervals calculated from 1000 bootstrap resamplings.

	CO	IR	SG	TT
CO	-	-0.0006	0.0011	0.0004
IR	-	-	0.0020	-0.0004
SG	-	-	-	0.0008
TT	-	-	-	-

CO–Forest restoration in Cosmópolis, SP; IR–Forest restoration in Iracemápolis, SP; SG–Natural remnant in Campinas, SP; TT—Natural remnant in Tietê, SP.

**Fig 3:**
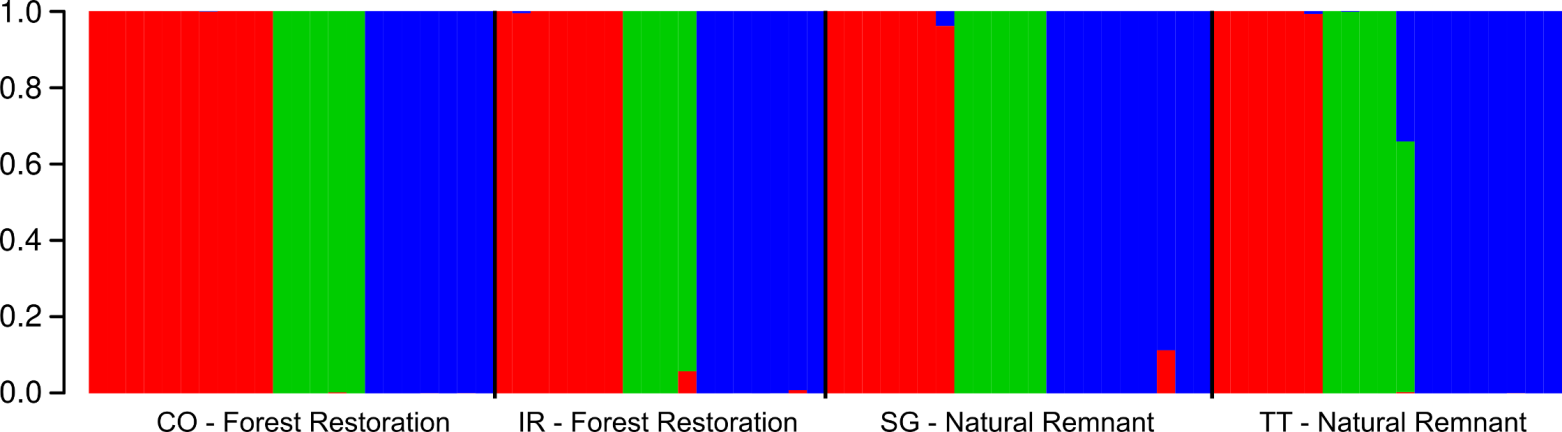
Bar plot of membership probability from Discriminant Analysis of Principal Components for *Casearia sylvestis* populations from restoration plantations and natural remnants in the Atlantic Forest of southeastern Brazil. Different colors represent tree different clusters found using BIC of DAPC. Each clusters is composed of samples from all pre-defined populations; thus, the genetic structure was not based on population subdivision. CO–Samples from forest restoration in Cosmópolis, SP; IR–Samples from forest restoration in Iracemápolis, SP; SG–Samples from natural forest in Campinas, SP; TT–Samples from natural forest in Tietê, SP.

Each of the groups generated based on Bayesian information criterion (BIC) was formed by samples from all populations (Fig 3), reinforcing the lack of genetic structure resulting from population subdivision.

We found no significant positive spatial autocorrelation at any distance interval (S2 Appendix). Moreover, no evidence of spatial genetic structure was detected in the forest fragments (Table 3). The regression slope of the kinship coefficient on the log of distance was not significantly different from zero (P > 0.05). Therefore, individuals located close to one another were not necessarily more genetically related than individuals separated by greater distances.

**Table 3:** Estimates of fine-scale spatial genetic structure for all *C*. *sylvestris* populations.

Pop.	β_log_	P-value (β_log_)	*F*_*1*_	*Sp*
CO	0.0038	0.1968	0.0646	-0.0041
IR	0.0007	0.8641	0.0144	-0.0007
SG	-0.0016	0.5165	-0.0065	0.0015
TT	-0.0025	0.4026	-0.0035	0.0025

β_log_−Regression slope of kinship coefficient on log of spatial distance; *F*_*1*_ –Kinship coefficient for the first distance class; *Sp—*Vekemans and Hardy’s (2004) estimator of fine-scale SGS intensity. CO–Forest restoration in Cosmópolis, SP; IR–Forest restoration in Iracemápolis, SP; SG–Natural remnant in Campinas, SP; TT—Natural remnant in Tietê, SP.

## Discussion

The value of research involving population genetics in a genomic scale using non-model species without previous knowledge of the genome size and complexity has been previously demonstrated in conservation genomics. With the advent of inexpensive sequencing and the advancement of techniques that reduce genome complexity, such as GBS, the study of genetic diversity and structure in natural populations can be based on hundreds of SNPs [56,57]. Here, we demonstrate the usefulness of this approach for restoration genetics.

We found no differences in the estimates of genetic diversity for *C*. *sylvestris* populations in natural remnants and forest restorations. The similar values between forest restorations and natural remnants are promising results for Brazilian Atlantic Forest conservation, because they indicate the effectiveness of forest restoration in maintaining the genetic diversity of the species, and consequently, the potential to self-perpetuate in the restored communities. Heterozygosity and allelic richness are good indicators of individual fitness in a population [15,58] and represent an initial estimate of the adaptive potential of a population to respond to environmental changes [59]. In previous studies developed by Cavallari et al. (2010) [60] on population relationships among *C*. *sylvestris* varieties in different ecosystems of Brazil, a similar pattern of genetic diversity was found in almost all populations. Thus, as measured by genetic diversity, the forest restoration plantations studied here were as conserved as forest remnants.

*Casearia sylvestris* is a pioneer species that is very abundant in disturbed fragments of the region and is highly dispersed by bats and birds, which may promote an intense gene flow between restoration plantations and remnants at the landscape level. Otálora et al. (2011) [24], studying the diversity and genetic structure of Mediterranean forests, suggest that as long as habitat quality and minimum connectivity remain among fragments, forest fragments can preserve the genetic diversity of original forests. In this context, forest restoration plantations can improve landscape connectivity and facilitate gene flow through seeds and pollen, which can mitigate the deleterious effects of inbreeding. In spite of the favorable results for genetic conservation, the studied populations had high F_IS_ values. Despite the mixed mating system of *C*. *sylvestris*, the differences found for these estimates might be related to the overwhelming level of forest fragmentation, as found in other studies conducted in fragmented landscapes, such as those of Vranckx et al. (2012) [61] and Dick et al. (2003) [62].

For the TT natural remnant, which is a highly fragmented forest, we expect that the reduction of population size in this type of forest and the isolation of the trees in the fragment contributed to the increase in F_IS_. Andrianoelina et al. (2006) [63], studying the effects of fragmentation on the genetic diversity of an arboreal species in Madagascar, suggest that an increase of F_IS_ in fragmented populations can be explained by high rates of self-fertilization caused by the isolation of adult individuals after population fragmentation.

The results for the general F_ST_ and the pairwise F_ST_ estimates among populations also suggested that the genetic diversity in natural remnants and forest restoration plantations was not structured through population subdivision. The DAPC results are consistent with the F_ST_ estimates and reinforce the potential gene flow among the studied forests and with other fragments of the region. Martins et al. (2016) [64] conducted an important study on the role of small natural remnants of Atlantic Forest for conserving genetic diversity of the tree *Copaifera langsdorffii*, and these authors suggest that small forest fragments preserve the genetic diversity that existed before fragmentation. Therefore, forest fragments are crucial for *in situ* species conservation and the maintenance of long-term genetic diversity in human-modified tropical landscapes. Moreover, by establishing restoration plantations with high genetic diversity, the risk of extinctions of tropical trees threatened by reproductive isolation in fragmented landscapes can be reduced.

To explain the absence of spatial genetic structure, we suggest that seed and pollen dispersal in these populations might play a role [65]. This result is consistent with previous discussions of genetic differentiation and structure for *C*. *sylvestris* populations in Brazilian Atlantic Forest fragments [66]. Populations of *C*. *sylvestris*, which is self-compatible, may be experiencing gene flow within and among populations in such a way that genetic diversity is uniformly distributed in space.

Sato et al. (2006) [67], studying the effects of gene flow on SGS for a riparian canopy tree species in Japan, suggested extensive gene flow occurs within and among populations. Furthermore, they argue that high levels of gene flow can increase the local effective size of a population and maintain genetic diversity. Bittencourt and Sebbenn (2007) [68] also suggest that pollen dispersal into forest fragments promotes genetic variation, reduces kinship and inbreeding, and increases the effective population size.

Therefore, we suggest that efforts are required to not only conserve large natural remnants but also the small forest fragments that are most of the remaining remnants in the Brazilian Atlantic Forest [2]. These remnants preserve the genetic diversity that might be important in the establishment and maintenance of new populations under future climate change scenarios. Additionally, we emphasize the importance of forest restoration as a way to connect forest fragments across the landscape, which will contribute to the long-term resilience of tropical forests embedded within human-modified landscapes in the current scenario of global environmental change.

## Supporting information

S1 Appendix Distribution and relative size of the fragments of Atlantic Forest used in the study of diversity and genetic structure of *Casearia sylvestris* Sw. populations of natural remnants and forest restorations. (PDF)

S2 Appendix Kinship correlograms (Loiselle et al., 1995) for *Casearia sylvestris* populations from restoration plantations and natural remnants in the Atlantic Forest of southeastern Brazil. (PDF)

S1 Dataset Coordinates of all *Casearia sylvestris* Sw. samples used in this study. (XLSX)

S2 Dataset SNP data of *Casearia sylvestris* Sw. samples used in this study. (VCF)
