# Supplementary material for: Genomic diversity is similar between Atlantic Forest restorations and natural remnants for the native tree Casearia sylvestris Sw
Source: PLoS One. 2018 Mar 7;13(3):e0192165. doi: 10.1371/journal.pone.0192165 (PMC5841640; doi:10.1371/journal.pone.0192165)

S1 Appendix - Distribution and relative size of the Atlantic Forest forest fragments used in the study of diversity and genomic structure of *Casearia sylvestris* Sw. populations of natural remnants and forest restorations.

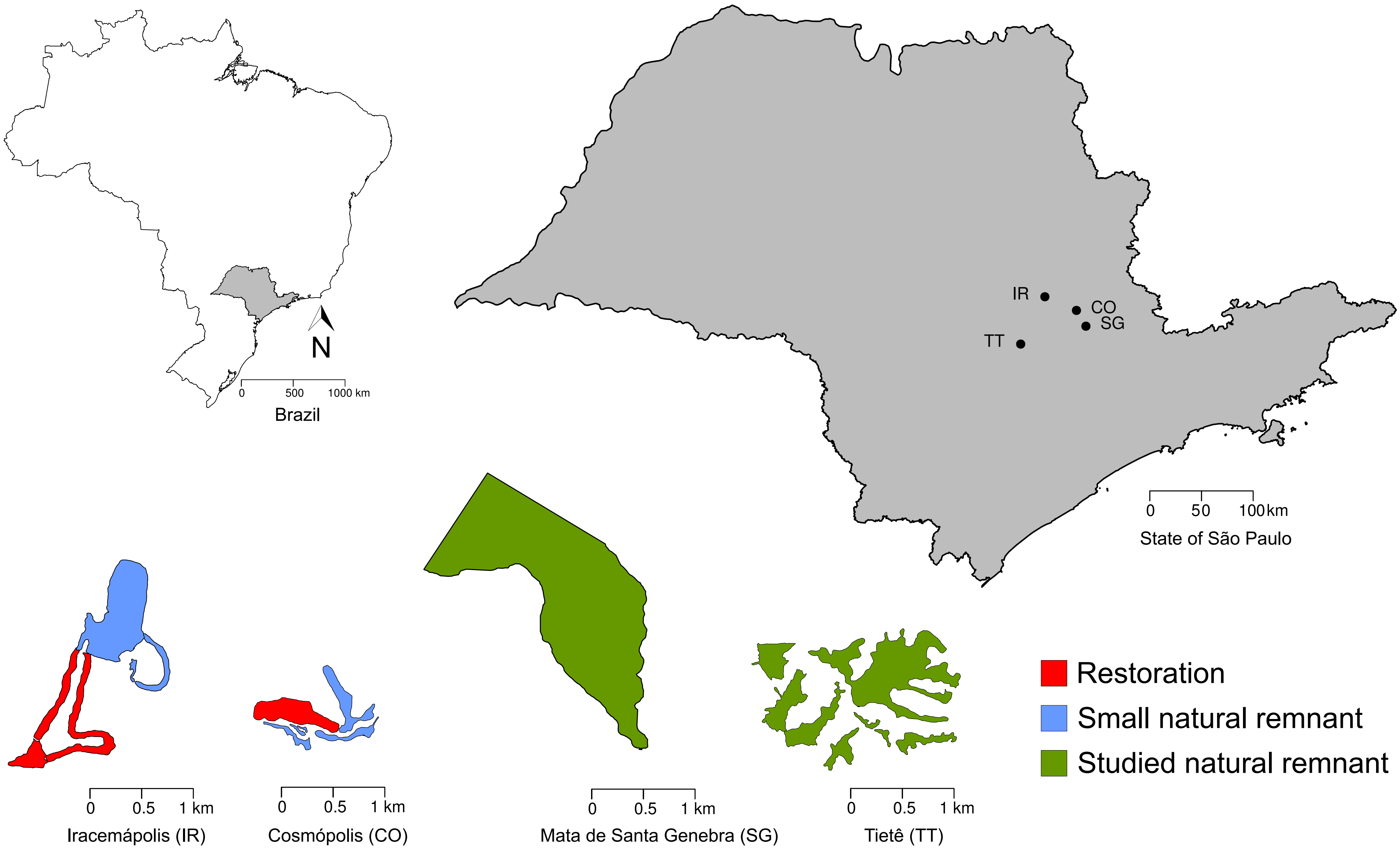

Supplement: S1 Appendix — (PDF) [file pone.0192165.s001.pdf]
