# Supplementary material for: Genomic diversity is similar between Atlantic Forest restorations and natural remnants for the native tree Casearia sylvestris Sw
Source: PLoS One. 2018 Mar 7;13(3):e0192165. doi: 10.1371/journal.pone.0192165 (PMC5841640; doi:10.1371/journal.pone.0192165)
Supplement: S2 Appendix — (PDF) [file pone.0192165.s002.pdf]

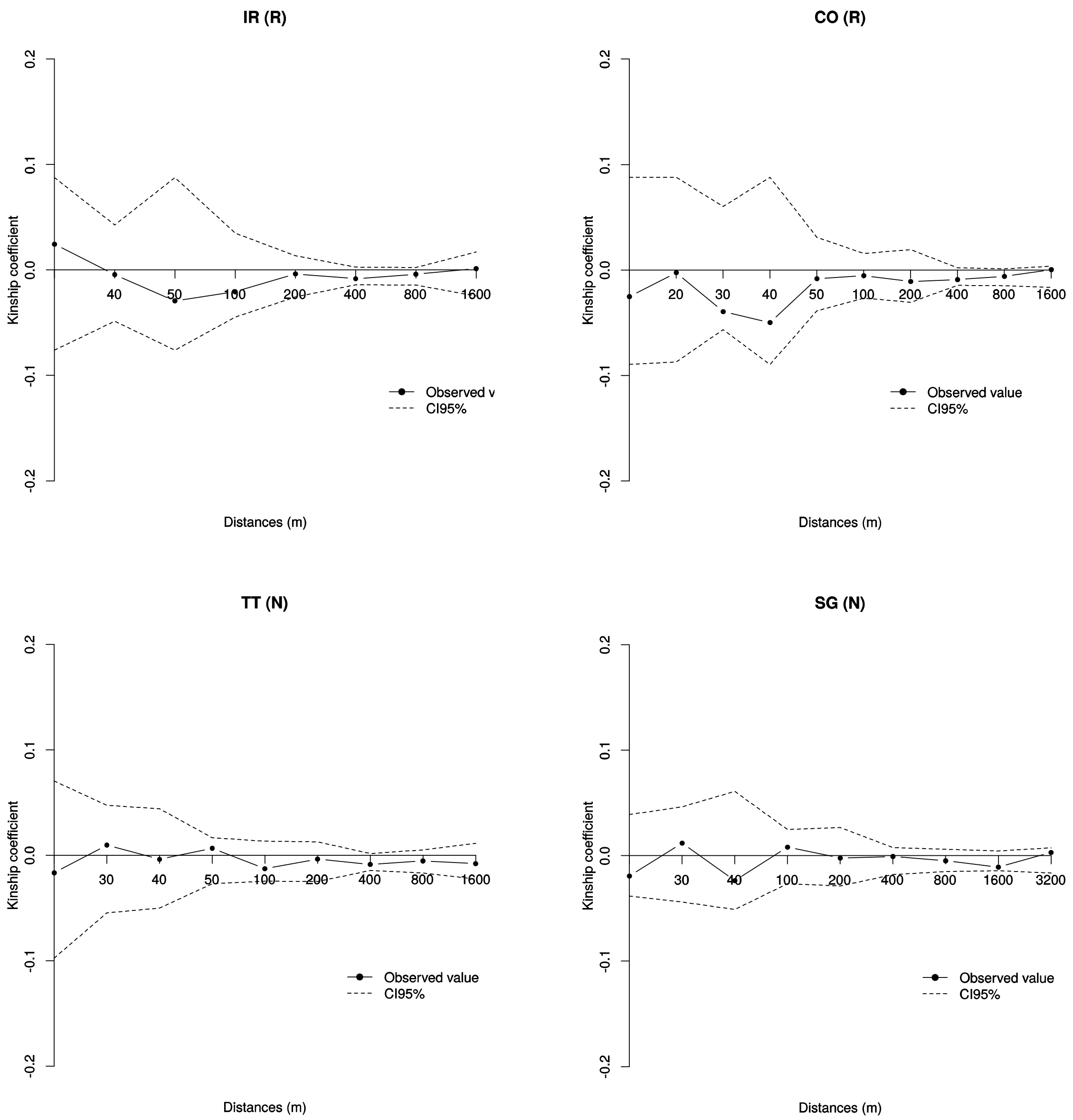

S2 Appendix – Kinship correlograms (Loiselle et al., 1995) for *Casearia sylvestis* populations from restoration plantations and natural remnants in the Atlantic Forest of southeastern Brazil. Filled circles correspond to mean kinship values in each distance class. Dashed lines are upper and lower 95% confidence intervals. CO – Forest restoration in Cosmópolis, SP; IR – Forest restoration in Iracemápolis, SP; SG – Natural remnant in Campinas, SP; TT - Natural remnant in Tietê, SP.
